# Sex Differences in Psychiatric Disease: A Focus on the Glutamate System

**DOI:** 10.3389/fnmol.2018.00197

**Published:** 2018-06-05

**Authors:** Megan M. Wickens, Debra A. Bangasser, Lisa A. Briand

**Affiliations:** ^1^Department of Psychology and Neuroscience Program, Temple University, Philadelphia, PA, United States; ^2^Neuroscience Program, Temple University, Philadelphia, PA, United States

**Keywords:** sex differences, glutamate, ADHD, Alzheimer's disease, schizophrenia, autism spectrum disorders (ASD), depression

## Abstract

Alterations in glutamate, the primary excitatory neurotransmitter in the brain, are implicated in several psychiatric diseases. Many of these psychiatric diseases display epidemiological sex differences, with either males or females exhibiting different symptoms or disease prevalence. However, little work has considered the interaction of disrupted glutamatergic transmission and sex on disease states. This review describes the clinical and preclinical evidence for these sex differences with a focus on two conditions that are more prevalent in women: Alzheimer's disease and major depressive disorder, and three conditions that are more prevalent in men: schizophrenia, autism spectrum disorder, and attention deficit hyperactivity disorder. These studies reveal sex differences at multiple levels in the glutamate system including metabolic markers, receptor levels, genetic interactions, and therapeutic responses to glutamatergic drugs. Our survey of the current literature revealed a considerable need for more evaluations of sex differences in future studies examining the role of the glutamate system in psychiatric disease. Gaining a more thorough understanding of how sex differences in the glutamate system contribute to psychiatric disease could provide novel avenues for the development of sex-specific pharmacotherapies.

## Introduction

Accumulating data indicate that disruptions in glutamate neurotransmission are a common underlying pathology in multiple psychiatric diseases including Alzheimer's disease (AD), major depressive disorder (MDD), schizophrenia (SCZ), autism spectrum disorder (ASD), and attention deficit hyperactivity disorder (ADHD) (Magri et al., 2008; Counts et al., 2011; Shimmura et al., 2011; Sokolow et al., 2012; Gray et al., 2015). Furthermore, these diseases all exhibit a sex bias, with increased prevalence of ASD and SCZ in men and increased prevalence of MD and AD in women (Fombonne, 2005; Noble, 2005; Markham, 2012; Mielke et al., 2014). Although little work has been done to elucidate baseline sex differences in the glutamate system, it is clear from work in these disease populations that sex differences must be considered. To promote a better understanding of these sex biases in disease along with sex differences in treatment response, we must first gain a better understanding of sex differences in the glutamate system. To date, very little work has been done to elucidate these differences. This review will focus on the sex differences in the glutamate system that have been revealed in clinical populations and preclinical studies of glutamatergic sex differences, highlighting how much more work is needed to obtain a clear picture of how sex differences in the glutamate system contribute to disease.

## The glutamate system

Glutamate is the primary excitatory neurotransmitter in the brain and it is essential for normal brain development and plasticity. Glutamate receptors come in two types, ionotropic ligand-gated ion channels and metabotropic, G-protein coupled receptors. These receptor subtypes can be even further subdivided. Currently there are 8 identified metabotropic glutamate receptors: mGluR1-8, and 3 identified ionotropic glutamate receptor subtypes: a-amino-3-hydroxy-5-methyl-4-isoxazolepropionic acid (AMPA) receptors, *N*-methyl-D-aspartate (NMDA) receptors, and kainate receptors. These receptor subtypes can be further divided based upon their subunit composition. AMPA receptors may be calcium-permeable or calcium-impermeable, depending on the absence or presence of the GluA2 subunit, respectively (Hanley, 2014). NMDA receptors are composed of two GluN1 and two GluN2 (or rarely GluN3 subunits). The four subtypes of GluN2 subunits (GluN2A-2D) confer functional diversity with each GluN2 subunit exhibiting unique biophysical, pharmacological and signaling properties (Paoletti et al., 2013; Sanz-Clemente et al., 2013; Wyllie et al., 2013; Ferreira et al., 2017). After being cleared from the synapse by excitatory amino acid transporters (EEATs), glutamate is converted to glutamine. As the levels of both glutamate and glutamine can be measured using proton magnetic resonance spectroscopy (MRS) in humans, many studies have examined these amino acids as potential biomarkers for psychiatric disease (Shimmura et al., 2011; Chiu et al., 2018; Sheikh-Bahaei et al., 2018).

## Sex differences in glutamate systems

### Baseline differences

The little work that has been done in humans to elucidate sex differences in the glutamate system has led to somewhat mixed results. MRS studies have demonstrated a slight increase in glutamate concentration within the parietal gray matter of men compared to women, while no differences were detected in the frontal gray or white matter or the basal ganglia (Sailasuta et al., 2008). However, when looking more carefully at specific brain regions, women seem to exhibit higher levels of glutamate compared to men. Specifically, women exhibit increased glutamate levels in the striatum and cerebellum compared to men (Zahr et al., 2013). There also appears to be increases in glutamate within the sensorimotor cortex and anterior cingulate cortex (ACC) of women (Grachev and Apkarian, 2000). Along with these studies examining glutamate within the brain, studies have also shown sex differences in serum glutamate concentration (Stover and Kempski, 2005; Teichberg et al., 2009). In contrast to the majority of studies examining glutamate in the brain, studies in blood have revealed higher glutamate concentrations in men compared to women (Zlotnik et al., 2011). As glutamate is present in many tissues in the body, these differences in serum glutamate may not reflect changes within the central nervous system (Shulman et al., 2006).

Sex differences in the glutamate system are more readily examined in rodent models. Several brain regions in rodents show sex differences in glutamate concentrations, including higher glutamate in the lateral hypothalamus and habenula of males and higher glutamate in the medial preoptic area of females (Frankfurt et al., 1984). Along with these overall sex differences in glutamate levels there are also changes in glutamate concentration across the estrous cycle (Frankfurt et al., 1984). These changes are brain region specific, with higher levels observed in the lateral septum during proestrus—the phase of the cycle where ovarian hormones are highest—compared to estrus; in the medial septum and diagonal band of Broca during proestrus compared to diestrus; and lower in the anterior hypothalamic area during proestrus compared to diestrus (Frankfurt et al., 1984).

Sex differences are also observed in synaptic glutamate signaling. Under basal conditions, female rats show larger hippocampal AMPR receptor synaptic responses, possibly due to enhanced phosphorylation of the GluA2 subunit (Monfort et al., 2015). However, this enhanced glutamate signaling may occlude further plasticity. Female rats show a reduction in the magnitude of tetanus-induced long-term potentiation (LTP) compared to male rats and this reduction is associated with a decrease in tetanus-induced phosphorylation of GluA1 (Monfort et al., 2015). As the phosphorylation of GluA1 AMPA subunits is involved in the insertion of GluA1-containing AMPR receptors into the synapse, this could reflect a mechanism for this diminished synaptic plasticity (Man et al., 2007). Along with these alterations in AMPA receptor signaling, sex differences also exist in NMDA receptor signaling. For example, NMDA antagonism increases prefrontal dopamine in male rats but decreases levels in females (Locklear et al., 2016). This may reflect a leftward shift in the dose response curve since females seem to be more sensitive to NMDA receptor manipulations. Female rats are more sensitive to excitotoxic damage following administration of an NMDA receptor antagonist, MK-801 (Wozniak et al., 1998) and exhibit a greater behavioral response to ketamine, an NDMA receptor antagonist (McDougall et al., 2017). This increase in NMDA sensitivity may be the result of increased receptor expression as female rats exhibit higher levels of both NR1 and NR2B NMDA subunits (Wang et al., 2015). Along with these changes in ionotropic glutamate signaling, there also appear to be basal sex differences in the metabotropic glutamate receptor system, with female rats exhibiting higher levels of mGluR2/3 and mGluR5 within the hippocampus along with increased mGluR5 in the prefrontal cortex (Wang et al., 2015). Steroid hormones may influence this overall increase in glutamatergic transmission. The neurosteroid, 17β-estradiol (E2) is known to potentiate excitatory transmission by increasing the probability of glutamate release in females (Smejkalova and Woolley, 2010).

### Changes across the lifespan

While relatively subtle sex differences in glutamate exist in healthy younger individuals, more dramatic sex differences seem to emerge with age. When examining glutamate levels in the brain across the lifespan, men exhibit a clear decline in glutamate from age 21 to age 70 within the basal ganglia and the parietal gray matter that is not present in women (Sailasuta et al., 2008). However, in the ACC, women show a more pronounced age-related decline (between ages 19 and 56) in glutamate levels compared to men (Hädel et al., 2013). Healthy men have been shown to have higher levels of glutamine (Gln) in the ACC, compared to healthy women (Tayoshi et al., 2009). In serum, women exhibit an increase in glutamate concentration as they age (from age 20 to 80), whereas men do not (Kouchiwa et al., 2012). Along with these age-related changes in glutamate levels, there appear to be changes in glutamate receptors as well. Over the course of aging (age 25 vs. age 70), men exhibit an increase in the distribution of mGluR1 in the cerebellum, parietal cortex, putamen, amygdala, and hippocampus (Sakata et al., 2017). Women do not show these aging-related differences in mGluR1 distribution (Sakata et al., 2017). Postmortem tissue analysis has demonstrated that glutamate related gene expression, including genes that code for glutamate receptors and trafficking proteins, decrease over the first 50 years of life within the prefrontal cortex (Choi et al., 2009). However, no studies have yet been adequately powered to detect normal sex differences in these effects nor have more advanced ages been examined. Nevertheless, studies on aging and disease provide us with some insight into potential differences.

Similar to the changes in the glutamate system that occur across the lifespan in humans, rodents also exhibit developmental changes in glutamate. Glutamate concentrations rise over the first 3 months of life in both male and female mice (Kulak et al., 2010). These changes in the glutamate system do not stop when animals reach adulthood. Glutamate concentrations decrease over the course of aging in the hippocampus, cortex, and striatum (Duarte et al., 2014). Although there were no sex differences in the total glutamate concentrations, the authors report a significant interaction between age and brain region in the ratio of glutamine/glutamate, which may reflect differences in glutamatergic transmission between neurons and glial cells (Duarte et al., 2014). Decreased levels of GluA1, GluN2A, and GluN2B glutamate receptor subunit levels over the course of aging (6 mo vs. 24 mos) have been correlated with poorer cognitive performance in male rats, but these studies have not been done in females (Ménard et al., 2015).

Taken together, although much more work is needed to fully understand sex differences in the glutamate system, there appears to be an overall increase in glutamate transmission in females. This increase may be subtle in young adulthood but during aging glutamate transmission decreases in males and the sex difference is amplified. These alterations in glutamate transmission at different ages could contribute to sex differences in incidence, symptomology, and treatment response for many psychiatric diseases. However, much more work is needed to examine differences within the glutamate system in different brain regions in males and females and determine whether there is in fact an overall increase in glutamate tone in females or if the differences are more subtle.

## Sex differences in glutamate systems in disease

### Alzheimer's disease

Alzheimer's disease (AD) is the leading cause of dementia and it is more likely to affect women than men, with nearly two-thirds of AD cases being women (Mielke et al., 2014). AD is characterized by accumulation of amyloid beta (Aβ) oligomers that are able to block glutamate uptake, leading to increased glutamate levels (Mattson et al., 1992; Domingues et al., 2007). This increased glutamate can lead to excitotoxicity and neurodegeneration. Dampening glutamate transmission can be helpful in the treatment of AD, as the non-competitive NMDA receptor antagonist memantine shows efficacy in the management of moderate-to-severe AD (Reisberg et al., 2003; Winblad et al., 2007). This increase in glutamate levels could more severely impact women with AD as they exhibit lower levels of GluA2-containing AMPR receptor subunits during late mild cognitive impairment compared to men at the same point in the progression of AD (Counts et al., 2011). Reduced levels of GluA2-containing AMPR receptor subunits could result in a greater proportion of GluA2-lacking, Ca^2+^-permeable AMPA receptors, and thus, increased vulnerability to excitotoxicity due to increased calcium conductance (Counts et al., 2011). To date, there are no studies that have examined whether glutamatergic drug treatments for AD exhibit similar effectiveness in men and women (Canevelli et al., 2017). Future work examining these sex differences in treatment response could provide insight into mechanistic differences in AD progression in men and women.

Just as sex differences are seen in patients with AD, sex differences are observed in AD phenotypes in mouse models of the disease. In the triple transgenic mouse model of AD (3xTg-AD), impairments in spatial memory and inhibitory avoidance tasks appear earlier in female mice than male mice (Clinton et al., 2007). Among 3xTgAD mice, both males and females show deficits in working memory, short-term memory, and increased anxiety-like behavior by 12 months of age, though female mutants show additional impairments in reference memory (Blázquez et al., 2014). This same early onset of cognitive deficits is also seen in two other mouse models of AD, tTa:APPsi mice, in which amyloid precursor protein (APP) expression is driven by the tetracycline transactivator (Melnikova et al., 2016) and APP(SW) mice which overexpress human APP (King et al., 1999). Furthermore, female mice exhibit greater deficits in cognitive function following overexpression of corticotropin releasing factor (CRF) in the presence of human APP compared to males (Bangasser et al., 2017). These differences in behavioral phenotypes are accompanied by differences in pathology. In another AD mouse model, the APP/PS1 transgenic line, female mice show an increase in plasma levels of amyloid protein with age, while males do not (Ordóñez-Gutiérrez et al., 2015). Female APP/PS1 mice also exhibit higher levels of parenchymal Aβ, particularly in the hippocampus, along with higher levels of phosphorylated tau and proinflammatory cytokines compared to male mutant mice (Jiao et al., 2016).

Building upon the work done in clinical studies, preclinical mouse models have found a role for glutamate in AD symptomatology. Learning deficits and amyloid plaque formation are among the AD symptoms implicated by disruptions in the glutamatergic system. Rats given a competitive NMDA receptor blocker showed deficits in reversal learning, yet no changes in the initial acquisition of a spatial memory task (Zhang et al., 2014), suggesting that NMDA receptors are at least partially involved in the learning deficits associated with AD. NMDA receptors have also been examined in mouse models. Treatment with memantine decreases amyloid plaque formation in APP/PS1-21 mice (Scholtzova et al., 2008). However, when treated with memantine, APP/PS1-21 mice performed similarly to WT controls in the object recognition test (Scholtzova et al., 2008). GluCEST and ^1^H MRS imaging of the APP-PS1 mouse model showed decreased glutamate levels throughout the brain (compared to WT controls), but the largest difference was observed in the hippocampus (Haris et al., 2013). This suggests that glutamate, and especially NMDA receptors, may be involved in the pathogenesis of AD (Monfort et al., 2015).

Furthermore, glutamatergic sex differences have been observed in preclinical models of AD. Reductions in glutamate within the dorsal hippocampus are seen only in male McGill-R-Thy1-APP rats and not females (Nilsen et al., 2014). Sex differences in AD development could be due to an interaction of glutamatergic systems with sex hormones. Estrogen is thought to play a protective role against cognitive impairments in female, and potentially male, rodents (Li C. et al., 2004; Frye et al., 2005; Carroll et al., 2007). It is hypothesized that estrogen is an underlying factor of sex differences in cognitive deficits following stress in rodents (Luine et al., 2007). After repeated stress, female rats show normal PFC glutamatergic transmission (Wei et al., 2014), suggesting that estrogen may be protective of PFC-mediated functioning. Furthermore, E2 treatment ameliorates Aβ-induced deficits in synaptic plasticity (Logan et al., 2011). However, as women age, their estrogen levels decline and this decline in estrogen may increase vulnerability (Barron and Pike, 2012). To date, the studies done in mouse and rat models of AD have not taken declining estrogen levels into account.

### Major depressive disorder

Women are nearly twice as likely as men to develop MDD and among those diagnosed with MDD, women experience more severe symptoms than men (Kornstein et al., 1995). Although the efficacy of SSRIs has focused the depression field on the serotonergic system, recent work on the efficacy of ketamine in treating MDD has led to increased interest in the glutamatergic system (Berman et al., 2000). Individuals with MDD have lower levels of both glutamate and glutamine in several brain regions including the ACC, dorsolateral prefrontal cortex (dlPFC), dorsomedial amygdala, and hippocampus (Auer et al., 2000; Michael et al., 2003a,b; Block et al., 2009). While the majority of studies have found this relationship, a few studies have not detected differences in glutamate metabolites (either glutamate or glutamine) in MDD (Binesh et al., 2004; Milne et al., 2009; Price et al., 2009). It is possible that some of the disparities in findings regarding glutamate levels and MDD are due to inconsistencies among participants between studies i.e., the ratio of men to women in the study and whether women were pre- or post- menopause (Gray et al., 2015). However, to date, none of these studies have examined potential sex differences in glutamate metabolite levels in MDD patients. Women with MDD have been shown to have higher levels of glutamate receptor gene expression postmortem, particularly in both AMPA and NMDA receptor subunit expression Additionally, women with postpartum depression exhibit an increase in prefrontal glutamate compared to healthy controls (McEwen et al., 2012). Thus, there is evidence for increased dysregulation in the glutamate system in women with MDD.

Similar to what has been seen in the clinical population, increased activity in the glutamatergic system has been connected to depression-like behavior in preclinical models. Male rats from the Flinders sensitive line (FSL), a model of depression, exhibit increased glutamatergic synaptic transmission in the hippocampus compared to controls (Gómez-Galán et al., 2013). However, female FSL rats exhibit higher levels of glutamate within the PFC compared to their male FSL counterparts (Kokras et al., 2009). Female rats also exhibit an increase in glutamate in the PFC in response to acute stress whereas males do not (Kokras et al., 2018). Furthermore, antidepressant administration increases cortical glutamate levels in both male and female FSL rats, while only increasing hippocampal glutamate in females (Kokras et al., 2009). Female rats expressing learned helplessness behavior, similarly would have increased glutamate, because they exhibit decreased glutamate uptake in the hippocampus, cortex, and striatum (Almeida et al., 2010). Furthermore, genetic alterations in the glutamate system can lead to depressive symptoms. Decreasing levels of vesicular glutamate transporter with a heterozygous knockout (VGLUT1^+/−^) leads to depressive-like behavior in mice (Tordera et al., 2011). However, chronic mild stress, another model of depression, leads to increased VGLUT1 levels in the hippocampus suggesting that bidirectional dysregulation of the glutamate system can be associated with depressive phenotypes (Garcia-Garcia et al., 2009).

Along with these broad differences in the glutamate system, preclinical models have revealed sex-specific alterations in the glutamate system in models of depression. Following prenatal chronic mild stress, male rats displayed higher expression of mGluR2/3, mGluR5, and NR1 in the prefrontal cortex; while female rats did not (Wang et al., 2015). Neonatal NMDA receptor blockade increases both physiological stress responsivity, CORT response, and anxiety-like behavior in the elevated plus maze in adult male mice, while female mice exhibit a decreased in anxiety-like behavior following the same treatment (Amani et al., 2013). Although the glutamate system of males appears more vulnerable to manipulations early in life, in adulthood, female mice are more sensitive to the antidepressant effects of ketamine, an NMDA receptor antagonist (Carrier and Kabbaj, 2013). Female mice exhibit a decrease in immobility in the forced swim test as well as an antidepressant response in the novelty suppressed feeding test at doses of ketamine that have no effect in males (Carrier and Kabbaj, 2013). These studies suggest that adult female mice have increased glutamate tone on NMDA receptors that may be leading to increased anxiety and depressive-like behaviors. This increased NMDA receptor tone may be responsible for the increased hippocampal dendritic spine density in females at baseline (Woolley et al., 1990; Shors et al., 2004). This idea is supported by work demonstrating that male and female rats exhibit opposite spine density changes in response to acute stress and these different responses are mediated by NMDA receptor activation (Shors et al., 2004). Further, this could provide a mechanism by which females are hyper-responsive to anxiety provoking stimuli in their environment.

### Schizophrenia

In contrast to AD and MDD, SCZ is more prevalent in men, with a male to female ratio of ~1.4:1.0 (Castle et al., 1993). Furthermore, men exhibit an earlier age of onset, greater symptom severity, and poorer response to treatment (Abel et al., 2010). Although there are many factors contributing to these sex differences, differences in the glutamatergic system are a critical component. Impairments in the glutamatergic system contribute to the pathophysiology of SCZ. (Olney and Farber, 1995; Goff and Coyle, 2001; Coyle et al., 2002; Tsai and Coyle, 2002; Javitt, 2007). However, this contribution appears to be different in men and women. For example, polymorphisms in different glutamate related genes increase the risk for SCZ in males and females. Multiple single-nucleotide polymorphisms (SNPs) in an X-linked gene coding for the AMPA receptor subunit 3, GRIA3, confer increased risk for the development of SCZ in females only (Magri et al., 2008). On the other hand, SNPs in the SAP97 gene that encodes a scaffolding protein involved in membrane targeting of glutamate receptors, is associated with an increased risk of SCZ in males but not females (Uezato et al., 2012).

Along with differences in genetic contributions, sex differences in glutamate related protein expression and metabolites have been found. Glutamine synthetase, an enzyme involved in the maintenance of glutamate levels, is upregulated in women with SCZ but not men (Martins-de-Souza et al., 2010). Additionally, women with SCZ exhibit higher levels of NMDA receptor density compared to men with SCZ (Nudmamud-Thanoi and Reynolds, 2004). NMDA receptor hypofunction is hypothesized to contribute to the pathophysiology of SCZ, therefore increased NMDA receptor density in women with SCZ could be protective and contribute to sex differences in symptomology (Leung and Chue, 2000; Coyle et al., 2002).

Examinations of sex differences in preclinical models of SCZ are few and far between. Much of the research on SCZ has focused on behavioral endophenotypes. Prepulse inhibition of startle (PPI), the reduction of startle produced by a prepulse stimulus, is diminished in patients with SCZ and can be easily modeled in animals (Swerdlow and Geyer, 1998). Female rats exhibit higher levels of PPI compared to males at baseline (Nozari et al., 2015; Zhang X. et al., 2015; Gogos et al., 2017). NMDA receptor antagonist, MK-801, decreases PPI in both intact and gonadectomized male mice whereas female mice only exhibit this decrease following ovariectomy (van den Buuse et al., 2017). This suggests that circulating hormones protect females against NMDA receptor mediated disruption of PPI. In support of this, estradiol treatment following ovariectomy blunts the ability of MK-801 to disrupt PPI (Gogos et al., 2012). Higher doses of MK-801 are able to disrupt PPI in females suggesting that NMDA receptors are still involved in the response in both sexes (Nozari et al., 2015).

In contrast to these static models of behavioral endophenotypes, developmental animal models of SCZ, such as the neonatal hippocampal lesion (nVHL) model, mimic the developmental progression of the disorder. The initial studies describing the nVHL model and the majority of those since then have utilized only the male pups, eliminating the ability to determine whether any sex differences exist (Lipska et al., 1993; Chambers et al., 1996; Flores et al., 1996; Goto and O'Donnell, 2002). An analysis of the literature revealed three papers that examined both males and females after nVHL. Overall, many of the behavioral effects of nVHL are similar in males and females, including deficits in working memory and increased locomotor response to novelty, MK-801 and amphetamine (Beninger et al., 2009; Bychkov et al., 2011). However, following nVHL, male mice exhibit hyperactivity in response to apomorphine, a non-selective dopamine agonist, whereas females do not (Bychkov et al., 2011). Further, following nVHL only male mice exhibit a decrease in phosphorylated extracellular signal-related kinase (pERK), mitrogen activated protein kinase (pMAPK), glycogen synthase kinase 3β (pGSK-3β), and protein kinase B (pAkt) in the accumbens and pERK within the PFC (Bychkov et al., 2011). In contrast, only female mice exhibit a decrease in pAkt and pMAPK in the dorsal striatum following nVHL (Bychkov et al., 2011). Along with these behavioral and molecular sex differences, there are also sex differences in the response to antipsychotics following nVHL. Clozapine can worsen working memory deficits in male nVHL mice whereas a floor effect may limit its effects in female nVHL mice (Levin and Christopher, 2006). However, control females are vulnerable to the memory dampening effects of clozapine whereas males are not (Levin and Christopher, 2006). Along with the nVHL model, neonatal administration of an NMDA receptor antagonist also induces SCZ-like behavior (Stefani and Moghaddam, 2005). However, the SCZ-like phenotypes are influenced by sex and hormonal status, with males and diestrous females exhibiting more consistent endophenotypes compared to proestrous females (Célia Moreira Borella et al., 2016). This could reflect a protective effect of estrogens, as levels of estradiol are highest during proestrus.

Studies utilizing mutant mice have also revealed sex differences that may be relevant to SCZ. The gene neuregulin1 (NRG1) confers an increased risk of SCZ and mutations in NRG1 lead to SCZ-like endophenotypes in mice (Gerlai et al., 2000; Stefansson et al., 2002, 2003, 2004; Li T. et al., 2004). However, there are sex differences in these phenotypes. While male neuregulin deficient mice exhibit deficits in object recognition memory and both contextual and cued fear conditioning, female Nrg1^+/−^ mice do not exhibit any cognitive deficits (Pei et al., 2014). Additionally, male Nrg1^+/−^ mice exhibited a decrease in the GABAergic markers, GAD67 and parvalbumin, while females did not (Pei et al., 2014). Although both male and female NRG1 mutant mice exhibit an increase in exploratory behavior, the specific elements of this behavior differed between males and female mutants (O'Tuathaigh et al., 2006).

Abnormalities in glutamatergic functioning have been associated with SCZ-like symptoms in animals. NMDA receptor hypofunction has been repeatedly cited as a component of SCZ and D-serine, an NMDA receptor co-agonist, may have therapeutic effects (Labrie et al., 2012). Accordingly, disrupting the glutamate system in a variety of ways, including neonatal NMDA antagonism (Stefani and Moghaddam, 2005) or deletion of AMPA GluA1 subunits (Procaccini et al., 2013), can lead to behavioral symptoms of the disease. Neonatal VHL rats also display disruptions in glutamate signaling, with reduced glutamate release in the PFC (Beninger et al., 2009). Furthermore, PCP and MK-801, NMDA receptor antagonists, have long been used to model the positive symptoms of SCZ (Moghaddam and Jackson, 2003). Perinatal treatment with PCP leads to deficits in spatial reference memory in male rats but not females (Andersen and Pouzet, 2004). Furthermore, these deficits were alleviated by treatment with D-serine, an NMDA co-agonist, suggesting that males may be more sensitive to disruptions of NMDA function than females (Andersen and Pouzet, 2004). Copy number variants (CNV) in the synaptic scaffolding molecular (S-SCAM), which controls synaptic AMPA receptor levels, have been linked to risk for SCZ. Transgenic mice with S-SCAM CNVs exhibit behaviors consistent with positive, negative, and cognitive symptoms of SCZ, as well as cellular and morphological abnormalities (Zhang N. et al., 2015). These mice also mimic the human condition because although both males and females show SCZ-like symptoms, male S-SCAM Tg mice generally exhibit more severe symptoms (Zhang N. et al., 2015). Taken together these findings suggest that increased glutamatergic tone in females may be protective and lead to differences in symptomology.

### Autism spectrum disorder

Similar to the sex bias seen in SCZ, ASD is more common in boys, affecting nearly four times as many boys as it does girls (Fombonne, 2009; Elsabbagh et al., 2012). Individuals with ASD have decreased levels of glutamate metabolites in the basal ganglia and ACC and these decreases are correlated with severity of ASD symptoms (Horder et al., 2013; Tebartz van Elst et al., 2014). In contrast to these decreases in glutamate metabolites in the brain, children with ASD have increased levels of glutamate in plasma and these levels also correlate with symptom severity (Cai et al., 2016). Despite the clear sex bias in the disease, to date, no studies have examined sex differences in metabolite levels (Ford and Crewther, 2016).

Similar to animal models of SCZ, animal models of autism focus on endophenotypes. In particular, autism-like behaviors in rodents have focused on deficits in social behavior. Healthy juvenile male mice exhibit more social exploratory behavior compared to juvenile females (Karlsson et al., 2015; Netser et al., 2017). Following prenatal valproic acid (VPA) treatment, an animal model of autism, male mice show impairments in social behavior in adulthood, while female mice do not (Kim et al., 2013). The prenatal VPA model also leads to male-specific deficits in sensorimotor gating, another phenotype of ASD (Anshu et al., 2017). Similar male-specific effects are seen in the telomerase reverse transcriptase overexpressing mice (TERT-tg). Male TERT-tg mice exhibit impaired social behavior, increased anxiety-like behavior, and lowered seizure threshold, while female TERT-tg mice do not (Kim et al., 2017). Maternal immune challenge also leads to male-specific deficits in social behavior in the contactin-associated protein-like 2 (Cntnap2) mouse model of ASD (Schaafsma et al., 2017). Individuals with ASD exhibit a decrease in striatal activation in response to social and non-social rewards (Scott-Van Zeeland et al., 2010). Male-specific deficits in reward learning are seen following 16p11.2 hemideletion, a gene that is disrupted in ASD (Weiss et al., 2008; Grissom et al., 2018).

Consistent with what has been seen in patients with ASD, preclinical studies demonstrate a clear role for the glutamate system in ASD-like behaviors. Extracellular glutamate concentrations in the lateral septum (LS) increase during social play for both male and female juvenile rats (Bredewold et al., 2015). In a mouse model of a common CNV found in ASD, ubiquitin protein ligase Ube3a, shows deficits in social interaction, impaired communication, and increased incidence of repetitive behaviors are accompanied by impaired glutamate synaptic transmission in male and female mice (Smith et al., 2011). A similar relationship is seen in both male and female Shank2 knockout mice. These mice show reduced social interaction and communication, impaired spatial learning and memory, and increased anxiety-like behavior, which are accompanied by reductions in NMDA receptor function (Won et al., 2012). Furthermore, restoring NMDA receptor function with D-cycloserine reversed the decreased sociability phenotype (Won et al., 2012). However, disruption of Shank3, another gene implicated in human ASD patients, leads to more pronounced reductions in glutamate transmission in male knockout mice and only juvenile males exhibit deficits in social behavior (Yang et al., 2012). As male mice exhibit higher levels of glutamate induced by social play compared to females, there may be sex differences in sensitivity to perturbations in the glutamate system (Bredewold et al., 2015). Furthermore, the increase in glutamatergic tone may be protective in females.

### Attention deficit hyperactivity disorder (ADHD)

Attention deficit hyperactivity disorder (ADHD) also shows a strong male bias, affecting nearly 3 times as many boys as girls (Cuffe et al., 2005). Traditionally mechanistic work on ADHD has focused on catecholamine function due to the therapeutic efficacy of stimulants. However, more recently the focus has shifted to the glutamate system due to data from genetic screenings implicating CNVs and SNPs in multiple glutamate receptor subtypes (Turic et al., 2004, 2005; Lesch et al., 2008; Mick et al., 2008; Elia et al., 2011). Furthermore, MRS imaging studies show increased glutamatergic tone in both the frontal cortex and striatum of ADHD patients and this is normalized by pharmacological treatment (Carrey et al., 2003; MacMaster et al., 2003). While no studies have examined male and female ADHD patients and made direct comparisons, female ADHD patients exhibit a positive correlation between ACC glutamate concentration and impulsivity (Ende et al., 2016). Glutamate may play a role in not only the pathology associated with ADHD but also the treatment response. Polymorphisms in NDMA receptor subunit genes predict better methylphenidate treatment response in children with ADHD (Kim et al., 2016). Notably, while studies discussed above controlled for sex, none of the published clinical studies have examined the influence of sex as an independent variable.

The majority of the work examining animal models of ADHD have either utilized only male mice to assess phenotypes (Archer et al., 1988; Kuwagata and Nagao, 1998; Kuwagata et al., 2004; Mergy et al., 2014) or in many cases where males and females were used data were collapsed preventing any examination of possible sex differences (Shaywitz et al., 1976, 1977; Pappas et al., 1980; Dell'Anna et al., 1991; Row et al., 2002). However, some studies utilizing the spontaneous hypertensive rat (SHR) model of ADHD have reported sex differences in behavioral phenotypes. Notably, while both male and female SHRs showed hyperactivity and sustained attention deficits, male SHRs exhibit greater impulsivity (Berger and Sagvolden, 1998). While there is evidence that male SHRs perform better on conditioned association tasks than female SHRs, this seems to reflect an increase in performance compared to controls in the males rather than a decrement in females (Bucci et al., 2008). Direct comparisons between controls and SHR males and females revealed attention deficits in male SHR rats that were not present in female SHRs, while both sexes exhibited increased inhibitory control and hyperactivity (Bayless et al., 2015). Along with these differences in behavioral phenotypes, animal models have also revealed sex differences in treatment response. Omega-3 polyunsaturated fatty acid supplementation lead to improved reinforcement-controlled attention in male SHRs while not affecting female SHRs (Dervola et al., 2012). These findings may be explained by the effects of sex hormones on fatty acid metabolism, particularly the low level of alpha-linolenic acid to docosahexaenoic acid metabolism in males (Dervola et al., 2012).

Just as alterations in the glutamate system have been implicated in human ADHD patients, animal models of ADHD also exhibit aberrant glutamatergic signaling. SHRs exhibit higher levels of glutamate-evoked norepinephrine release and slower AMPR receptor internalization within the hippocampus compared to controls (Howells and Russell, 2008). Given that there is evidence for increased extracellular glutamate within the hippocampus, these downstream effects could be even greater than they appear (Sterley et al., 2016). This increase in extracellular glutamate may occur outside the hippocampus as well. SHR males have heightened levels of evoked glutamate release in the PFC and striatum compared to controls (Miller et al., 2014). Furthermore, manipulations of the glutamate system can lead to ADHD-like behaviors. Infusion of the NMDA antagonist, 3-(R)-2-carboxypiperazin-4-propyl-1-phosphonic acid, into the mPFC of rats leads to increased impulsivity and compulsivity (Pozzi et al., 2011). Despite this link between glutamate and ADHD behavioral phenotypes and the observed sex differences in preclinical models, differential roles of glutamate or alterations in the glutamate system have not been examined in sex-specific manner.

## Conclusion

These studies clearly demonstrate a role for dysregulation in the glutamate system in sex biased psychiatric diseases. The little data that are available suggest that females have increased glutamatergic tone compared to males and this can increase vulnerability in some cases and be protective in others. However, very little work has been done to elucidate potential sex differences in the glutamate system either at baseline or in the disease state (see Table 1). Although more imaging and postmortem tissue analysis in clinical populations would be insightful, a basic understanding of sex differences in glutamate signaling is needed. To achieve this, more preclinical studies aimed at determining sex differences are warranted. After a fundamental understanding of baseline differences is reached, examination of how dysfunction in the glutamate system can contribute to psychiatric disease would be more informative. As the majority of preclinical work has been done either only in male rodents or studies that have been underpowered to examine sex differences, much of what we know about glutamate system function and psychiatric disease may only apply to males. The examination of how glutamate dysfunction differentially affects males and females could lead to novel avenues for therapeutic development in these sex biased diseases.

**Table 1:** Sex differences in glutamate systems in disease.

**Clinical studies**	**Symptomology incidence**	**Serum glu**	**Brain glu**	**Glu receptor activity/expression**	**Response to glu drugs**
Baseline	N/A	♂> ♀	♀> ♂	–	N/A
Aging	N/A	↓ ♀	↓ ♂	↑ ♂ mGlu1	–
AD	↑ ♀	–	–	↓ ♀GluA2	–
MDD	↑ ♀	–	–	↑ ♀ ✖♂	–
Schizophrenia	↑ ♂	–	–	↑ ♀ ✖♂	–
ASD	↑ ♂	–	–	–	–
ADHD	↑ ♂	–	–	–	–
**Preclinical**	**Glutamate concentration**	**Glu receptor expression**	**Glu transmission**	**Synaptic plasticity**	**Response to glu drugs**
Baseline	–	♀> ♂	♀> ♂	♂> ♀	N/A
Aging	↓ ♂↓ ♀	↓ ♂✖♀	–	–	–
AD	↓ ♂	–	–	–	–
MDD	↑ ♀ > ↑ ♂	–	–	–	↑ ♀ ✖♂
Schizophrenia	–	–	–	–	↑ ♂ ✖♀
ASD	–	–	↓ ♂=↓ ♀	↓ ♂=↓ ♀	–
ADHD	–	–	–	–	–

*This table summarizes the studies that have examined sex differences to date. The rows for each disease state reflect changes from control or baseline in each sex, respectively. The line symbolizes that no studies have examined sex differences for the variable in a given disease state. There may be data for changes in one sex or data collapsed across sex that indicate a change from baseline but no studies have explicitly examined sex differences*.

## Author contributions

MW, DB, and LB contributed to the conception and design of this review, and edited and revised the manuscript. MW and LB wrote the manuscript. MW, DB, and LB have seen and approve of the final version to be published.

### Conflict of interest statement

The authors declare that the research was conducted in the absence of any commercial or financial relationships that could be construed as a potential conflict of interest.
